# Application of image recognition technology in pathological diagnosis of blood smears

**DOI:** 10.1007/s10238-024-01379-z

**Published:** 2024-08-06

**Authors:** Wangxinjun Cheng, Jingshuang Liu, Chaofeng Wang, Ruiyin Jiang, Mei Jiang, Fancong Kong

**Affiliations:** 1 Center of Hematology, The First Affiliated Hospital, Jiangxi Medical College, Nanchang University, Nanchang, 330006 China; 2 Queen Mary College, Nanchang University, Nanchang, 330006 China; 3 Department of Clinical Laboratory, The First Affiliated Hospital, Jiangxi Medical College, Nanchang University, Nanchang, 330006 China

**Keywords:** Blood smear, Blood cells, Image recognition, Deep learning

## Abstract

Traditional manual blood smear diagnosis methods are time-consuming and prone to errors, often relying heavily on the experience of clinical laboratory analysts for accuracy. As breakthroughs in key technologies such as neural networks and deep learning continue to drive digital transformation in the medical field, image recognition technology is increasingly being leveraged to enhance existing medical processes. In recent years, advancements in computer technology have led to improved efficiency in the identification of blood cells in blood smears through the use of image recognition technology. This paper provides a comprehensive summary of the methods and steps involved in utilizing image recognition algorithms for diagnosing diseases in blood smears, with a focus on malaria and leukemia. Furthermore, it offers a forward-looking research direction for the development of a comprehensive blood cell pathological detection system.

## Introduction

Image recognition, denoting the utilization of computational mechanisms for the processing, analysis, and comprehension of visual data, enabling the identification of diverse patterns, targets, and entities, epitomizes a practical implementation of deep learning algorithms [1]. The traditional process of image recognition is divided into five steps: image acquisition, image preprocessing, image segmentation, feature extraction, and image classification. In recent years, the digitalization process in the field of medicine has been continuously advancing, and image recognition technology, as a tool, is gradually being used to optimize existing medical techniques. Image recognition has been extensively studied in the application of in vitro diagnostic products, including various microscopic images and imaging images, in which deep learning methods have been widely used [2–4].

Blood smear examination is an important auxiliary diagnostic method for diseases. Blood cell images contain three types of cells: leukocytes, erythrocytes, and platelets [5]. Through identifying and counting these three types of cells in an image, physicians can obtain information about individual health status and disease severity, which is helpful in determining treatment regimens or adjusting treatment strategies. The test results are relevant to conditions such as leukemia, liver diseases, and can also provide early indications of certain tumors, enabling early detection and treatment [6, 7]. The traditional method of blood cell examination involves manual inspection using a microscope. Initially, a blood sample is extracted from the patient's body and prepared into a blood smear [8]. Subsequently, staining methods such as Wright staining and Giemsa staining are employed for processing [9]. The prepared slide is then placed under a microscope, and various types of cells in the blood are identified through visual observation. Obviously, this method has disadvantages, including time-consuming, labor-intensive and inefficient. It is worth noting that the traditional method is prone to counting errors, leading to misdiagnosis. Discrepancy between physician's experiences is an important reason for misdiagnosis. [10, 11].

Currently, the most widely used method for blood cell analysis is the hematology analyzer. These instruments offer the advantages of convenience, efficiency, and high accuracy of test results [12, 13]. However, the analyzers are limited by high development costs, inability to obtain cell images, and lack of capability to assess cell morphology. Fortunately, a new type of analytical instrument utilizing image processing algorithms for cell detection has emerged on the market, such as the RWD C100 cell counter which employs clustering algorithms for cell identification and counting [14], and the Countess series cell counters by Thermo Fisher Scientific which utilize neural network algorithms for cell counting [15]. The use of appropriate image processing algorithms for cell detection is becoming increasingly mainstream. Analyzing cell images can better reveal the pathogens and etiology, playing an increasingly important role in disease prevention and treatment guidance. Accurate detection and identification of various blood cells constitute the crux of cellular image analysis, yet this remains a challenge [11]. Due to the diverse morphologies of cells in microscopic cell images and the variety of cell types, it remains quite challenging to use image processing algorithms to identify and count cells. The use of computer graphics to assist doctors in observing the morphological features of blood cells has become a major trend [16]. In recent years, deep learning methods have experienced significant advancements, giving rise to algorithm models such as convolutional neural networks (CNN). These models have been widely applied in the fields of classification, recognition, and segmentation, showcasing notable advantages such as a straightforward process and high recognition rates. As a result, they also hold vast potential for application in medical image detection [17–19]. Neural networks have also been widely applied in cell detection and counting tasks [20]. Due to their autonomous learning characteristics, neural networks are commonly used and effective processing methods in image recognition. Furthermore, neural network learning can optimize the cumbersome steps in traditional image recognition, to some extent, avoiding the biases brought about by manual selection in traditional image processing, thus achieving better fault tolerance while improving recognition efficiency.

The process of using image recognition technology to assist the pathological diagnosis of blood smears should be divided into five steps: sample processing and collection → preprocessing → detection and segmentation → feature extraction and selection → assessment and classification. Segmentation refers to isolating various types of cells from blood images based on different characteristics, playing a pivotal role in the diagnosis, planning, and guidance of diseases [21]. Feature extraction involves selecting attributes that distinguish each cell type based on unique color, shape, texture, and other features [22], significantly enhancing the performance of diagnostic models [23]. Image classification entails the use of appropriate algorithms for cell identification tasks based on extracted features, yielding results [24]. The recently resurgent neural network methods can perform segmentation or detection of blood cells at the pixel level, furthermore, convolutional neural networks can be considered as feature extraction methods that are robust to translational variance [25]. Neural networks, combined with classifiers, can also carry out classification tasks, thereby reducing the biases introduced by manual selection methods at various stages. In view of the prospect of the wide application of image recognition technology in the pathological diagnosis of blood smears and for the sake of the further development of subsequent novel analytical instruments in future, this paper will provide a systematic review of the application of image recognition technology in the detection of blood smears.

## Blood smear microscopic image detection

The microscopic image diagnosis of blood smears has long relied heavily on pathologists. The ability to accurately diagnose diseases often depends on the professional knowledge and experience, which introduces a certain level of complexity and subjectivity [10, 26]. As early as 2011, scholars attempted to explore the application of image recognition technology in the diagnosis of malaria from blood smears. Prasad Keerthana et al. developed a malaria diagnostic decision support system (DSS) using color image analysis technology [27]. Since hematologists must examine approximately 100 to 300 microscopic views of Giemsa-stained thin blood smear images to detect malarial parasites and assess the degree of infection, the system extracts suspicious areas of the blood smear and detects parasites in the view [28]. It then communicates this information to remote experts to obtain accurate diagnoses and treatments [27]. Diseases typically detected through the observation of blood smears encompass various blood system disorders and parasitic diseases, including leukemia and malaria [29]. With technological advancements, in 2017, some studies attempted to reduce manual intervention and achieve intelligent detection. They employed CNN algorithms to read images of blood smears, extract different features, and construct models capable of identifying malarial parasites within red blood cells, thereby achieving the goal of automated diagnosis [30]. The widespread application of convolutional neural network algorithms in the image detection of blood smears has commenced, particularly for diseases commonly diagnosed using blood smear methods, such as malaria and leukemia.

Leukemia is a malignant progressive disease, characterized by an increase in the number of white blood cells in the blood and/or bone marrow [31]. Leukemia can be classified into two major categories based on the speed of progression and the type of cells affected: acute and chronic leukemia [32]. Acute leukemia is characterized by the abnormal proliferation of immature white blood cells in the bone marrow, leading to incomplete cellular development [33]. In contrast, chronic leukemia involves the abnormal proliferation of mature white blood cells in the bone marrow; these cells appear more mature morphologically but are functionally defective [34]. Among acute leukemias, the most common types include acute lymphoblastic leukemia (ALL), acute myeloid leukemia (AML), and acute promyelocytic leukemia (APL) [35]. ALL arises from the abnormal proliferation of lymphocytes, whereas AML results from the abnormal proliferation of immature myeloid cells in the bone marrow [36, 37]. Acute promyelocytic leukemia is a subtype of myeloid leukemia, distinguished by the presence of a large number of promyelocytes among the immature cells [38]. Chronic leukemias encompass chronic myeloid leukemia (CML), and chronic lymphocytic leukemia (CLL) [39]. Chronic myeloid leukemia is caused by the abnormal proliferation of mature granulocytes in the bone marrow, while chronic lymphocytic leukemia arises from the abnormal proliferation of lymphocytes [40]. Leukemia was the leading cause of the proportional disability adjusted life year (DALY) burden in adolescents in 2019 [41]. It was also the 15th most commonly diagnosed cancer and the 8th leading cause of cancer-related deaths globally in 2020, accounting for 3.1% of all cancer deaths [42]. The death-to-case ratio for leukemia was as high as 74% 20 years ago, reflecting poor detection and prognosis of leukemia in many parts of the world. Compared to that time, there has been a 58% increase in leukemia cases and a 40% increase in mortality [42]. Although this demonstrates some progress in the detection and prognosis of leukemia, with a decrease in the mortality rate, it is important to note that leukemia remains a highly prevalent disease. Furthermore, currently, especially in developing countries, healthcare systems and technologies have led to insufficient diagnosis of leukemia [43, 44]. Currently, the automated diagnosis of leukemia has been globally recognized, with the automated diagnosis of ALL being the most advanced among all subtypes of leukemia. Sarmad Shafique et al. summarizes the procedure of the computer-aided system from four phases, namely, preprocessing, segmentation, feature extraction, and classification. A large number of studies have been conducted to explore the detection of ALL from various directions, including methods utilizing deep learning [45], methods based on genetic algorithm optimization [46], methods based on convolutional neural networks [47], and integrated detection methods incorporating modern intelligent medical Internet technologies [48]. ALL, caused by bone marrow dysfunction, is the most commonly diagnosed childhood cancer worldwide [49], with survival rates decreasing with increasing age at diagnosis [50]. Additionally, research has revealed that image recognition technology holds significant potential for the precise diagnosis of other types of leukemia and is continuously evolving. The experimental evidence has confirmed the potential application of this technology in AML, CML, and CLL [51–53]. The initial diagnosis of leukemia involves the manual assessment of stained blood smear microscopic images. However, hematologists face challenges in using blood smears to determine a patient's condition due to the time-consuming, inaccurate, and subjective nature of the process [54]. As a result, numerous computer-aided diagnostic systems have been developed to autonomously identify leukemia cells within blood images. Currently, the common computer-aided diagnostic systems fall into two major categories: those based on deep learning technology and those based on machine learning algorithms [55]. They are capable of efficient data processing, automated feature extraction, classification and assessment, thereby significantly enhancing the accuracy and efficiency of diagnosis [56, 57].

Malaria is a severe disease caused by parasitic infection, with five known parasites capable of causing human malaria. Among them, the most deadly is the malignant malaria parasite, responsible for almost all malaria-related deaths [58]. Nearly half of the world's population is at risk of contracting malaria, and despite advances in malaria control in developed countries in recent years, the disease remains prevalent in other global regions, such as Africa [59]. According to the World Health Organization (WHO), there were approximately 241 million cases of malaria globally in 2020, with an estimated 627,000 deaths [60]. Rapid and accurate diagnosis of malaria is crucial for alleviating the burden of the disease [61, 62], if left untreated within 24 h of symptom onset, malaria may rapidly progress to a life-threatening illness [63]. Furthermore, monitoring of malaria plays an indispensable role in the prevention, control, and elimination of the disease, especially in the elimination phase [62, 64, 65]. Malaria monitoring involves continuous and systematic collection, analysis, and interpretation of malaria-related data, as well as the use of this data in planning, implementation, and evaluation of public health practices to assist countries in understanding disease patterns, designing effective health interventions, and evaluating the measures [66]. Monitoring indicators typically include 11 transmission indicators such as clinical diagnosis positivity rate determined by microscopic examination of blood smears, entomological inoculation rate, and clinical malaria incidence or annual parasite index. The sensitivity and specificity of the clinical diagnosis positivity rate determined by microscopic examination of blood smears have been well evaluated [67, 68], indicating that microscopic examination of blood smears remains the gold standard for diagnosing and monitoring malaria. Efforts have been made to use machine learning techniques to assist in the detection of malaria. For the blood smear analysis system, comprehensive disease detection is the key to future development. The integration of corresponding detection methods on the same machine enables the full intelligence of blood smear pathology diagnosis. The following text will mainly focus on summarizing the overall auxiliary testing process for malaria and various types of leukemia (with a primary focus on ALL), and propose a comprehensive and systematic future construction plan through comparative analysis.

### Sample collection

#### Preparation of blood smears

Giemsa staining is commonly used to differentiate cell nuclei from parasites, with the advantage of low cost, low contamination, and precise results [69, 70]. Wright staining is widely used for differential counting of leukocytes and platelets [71, 72]. Myeloperoxidase (MPO) is a peroxidase that can be applied to the diagnosis of AML and APL [73]. Sudan black B staining can be used to differentiate ALL from AML [74]. Terminal deoxynucleotidyl transferase (TdT) is an immunostaining method used for the examination of ALL and lymphoma [75, 76]. Field staining can reduce staining time, but the consistency of results is slightly lower than traditional Giemsa staining, and it can only be used for thick smears to specifically differentiate basophilic and acidophilic cells [77, 78]. In comparison, Leishman staining is suitable for thin smears and demonstrates superior sensitivity to malaria parasites [79]. Additionally, Leishman staining has good contrast, especially between the cell nucleus and cytoplasm [80]. Compared to Leishman staining, New methylene blue (NMB) staining for detecting malaria parasites is a simpler process with lower cost, but it has some toxicity [81, 82]. Acridine orange (AO) staining is considered a rapid alternative to Giemsa staining [70, 83], but its sensitivity and specificity are still insufficient, with issues such as inconsistent staining and short-lived fluorescence [84]. Therefore, if one wants to construct a comprehensive testing system, the Wright-Giemsa stepwise combined staining method for blood smears has a higher comprehensive benefit [85].

#### Collection of microscopic images

The microscopic analysis of stained smears is one of the most operator-dependent and time-consuming activities in clinical microbiology laboratories [86]. The method of collecting microscopic images after staining blood smears also greatly influences the subsequent algorithms' recognition of cells. If one wants to improve the accuracy of actual clinical testing, the standardization of sample preprocessing and collection is particularly important.

Traditional methods of microscopic image collection typically involve capturing images of smears under a microscope using a digital camera, which is time-consuming and lacks standardization [87]. With continuous breakthroughs in optical imaging technology, fully automated scanning microscopes are currently the tool to address the challenge of cell-level image acquisition. The development of microscopic image acquisition devices has been iterative, from the initial realization of image acquisition at lower magnifications [88] to the recent updates of cell-level microscopic image devices [89]. Breakthroughs in hardware technology in other fields are important groundwork for promoting the construction of intelligent medical diagnostic assistance systems (Table 1).

**Table 1:** Methods employed in the various stages of image recognition detection of different types of leukemia and malaria

	Pretreatment	Segmentation	Feature extraction	Classification	Number of images	Rate of accuracy	
*Leukemia*							
Krishna Kumar Jha et al.		Entropy-based mixture model	Color	Chrono-SCA-ACNN		99%	[93]
Atteia Ghada et al.	Enhance			CNN	368	99.82%	[121]
Krishna Kumar Jha et al.	Mixed model based on MI	Active contour model, fuzzy C-means algorithm	Texture	SCAbased Deep CNN classifier		68.2%-98.7%	[112]
Das Biplab Kanti et al.	Enhance	LUV color transform, adaptive threshold	Texture and Morphology	GFNB classifier		96%	[100]
Ghada Atteia		Color threshold-based segmentation under HSV color space	Characteristic metastatic characteristics (FTFs)	SVM and EL classifier	3562	96%(SVM);95.6%(EL)	[119]
E. RABIZADEH	Rotation, Flip, Contrast change, Luminosity change, Focal length adjustment	GANs	Morphology	CNN	10 00	87.7% ~ 100%	[116]
Yunfei Liu	Enhance, Flip, Affine transformation, Gray scale transformation, and Color dithering	Weakly supervised learning	The coarse granules, chromatin organization of the nucleus	WT-DFN	12,994	83.41% ~ 91.50%	[26]
Priyanka Rastogi	Enhance, Translate, Flip		Morphology	SVM、 XGB、 RF、 ETC	60,000	92.3%(LeuFeatx + SVM) 、92.67%(LeuFeatx + XGB) 、95.76%(LeuFeatx + RF) 、96.15%(LeuFeatx + ETC)	[53]
Tusneem		CM-YK moment location-feature fusion extraction framework	Morphology	DCAE-CNN deep learning model	18,365	99.70%	[52]
Laura Boldú	Enhance, Flip		Morphology	CNN classifier	16,450	89.5% ~ 100%	[52]
Niranjana Sampathila	Improve the contrast	HSI color space and thresholding	Color	CNN	10,661	95.45%	[160]
John-William Sidhom	Enhance and eliminate batch effect		Morphological features of genomic imprinting	MIL			[161]
Laura Boldú et al.		Color fuzzy clustering, watershed transformation	Color	LDA classifier	442	94%	[51]
*Malaria*							
Das Dev Kumar et al.	Illumination correction and noise reduction	Transformation of watershed	Morphology	Bayes learning、SVM		(Bayesian approach) 84%; SVM 83.5%	[94]
Dahou Yang et al.	Noise reduction (Total generalized variation (TGV))	Local adaptive threshold	Morphology	SVM			[70]
Dhanya Bibin et al.		Level set	Color, Texture	Deep learning based on DBN		Up to 96.41%	[79]
Angel Molina et al.		Threshold segmentation, Watershed transformation	Morphology	Deep learning based on CNN	6415	99.5%	[111]
Angel Molina	Enhance, Smooth and Reduce noise	Transformation of watershed	Morphology, Color	SVM, KNN, LDA, Random forest and Gaussian Naive Bayes	15 660	0.977	[122]
Jung Yoon	Noise reduction and Enhancement (median filtering and adaptive histogram equalization techniques)	Connected component labeling method	Morphology			Better	[97]
OSMAN AKCAKIR	Strengthen	Edge detection	Geometric/phase Morphology and Texture features	Random forest model, VGG16 deep learning model	17,440	91% (Random forest model) 、98% (VGG16)	[93]
Amal H. Alharbi	Normalization, Noise reduction, Data enhancement	Hough transform	Morphology	Pretrained convolution neural network model (VGG-19)	27,558	97.14%	[162]
Geng Wang	Noise reduction, Data enhancement, and Standardization		Morphology	YOLOv7 model	12 708	> 96.8%	[113]
Dilber Uzun Ozsahin	Enhance and de-noise	Mask R-CNN、ResNet50、U-Net andFaster R-CNN	Morphology	CNN model	564	96.97%,	[109]
Amal H. Alharbi	Enhance and Smooth	Deep learning semantic segmentation method	Morphology	Support vector machine, XG-Boost model, and Neural network model	27,500	94% (support vector machine), 90% (XG-Boost model), 80% (neural network model)	[163]

This table summarizes the methodologies employed in the various stages of image recognition detection for diverse types of leukemia and malaria in recent years

Smith Kenneth P. et al. studied a method based on automatic image acquisition and CNN for automatic classification in Gram staining. Using an automatic microscope platform, slides were scanned with a 40 × objective lens, generating images of sufficient resolution for interpretation [90]. Hu Qinglei et al. have recently invented a digital microscopic imaging system for biological samples. The microscopic imaging system can achieve direct imaging of transparent or semi-transparent samples without actual staining, addressing the limitations of sample staining in traditional microscopic inspection methods, simplifying the inspection steps, and shortening the inspection time [91]. This invention may provide new thinking for achieving fully automated detection in future.

### Data preprocessing

When applying image recognition technology for the diagnosis of leukemia and malaria in blood smears, the preprocessing work covers a series of critical steps from data acquisition to data enhancement to ensure the acquisition of high-quality image data [92, 93]. First, the blood smear collection process was strictly controlled to ensure consistency. Second, images were captured under uniform illumination using a high-resolution camera, and illumination correction [92] and normalization [94] were performed to eliminate variations in brightness and color. This also eliminates the effects of batch variations during image acquisition, thereby improving the stability and accuracy of the model. Noise reduction algorithms are used to eliminate random noise, such as generalized Gaussian noise reduction, to reduce image noise, making the images more suitable for model training [95]. Smoothing noise reduction can make the images clearer [96]. Median filtering and adaptive histogram equalization techniques are employed to reduce noise in the images and enhance image contrast [97]. Morphological processing improves cell contours and removes noise, while feature enhancement techniques highlight key features such as cell nuclei, cytoplasm, and parasite nuclei [98]. Data augmentation, through operations such as image rotation, flipping, and translation, is used to increase sample diversity and improve the model's generalization capability [99]. Das Biplab Kanti also proposed a preprocessing stage in the experiment, where automatic enhancement of input blood smear images is performed using a Gini index-based Fuzzy Naive Bayes classifier (GFNB) [100]. The preprocessing steps are crucial, significantly enhancing the quality and consistency of the data, laying a solid foundation for subsequent image analysis and machine learning model training to improve the accuracy and efficiency of blood smear pathological diagnosis.

### Detection and segmentation

In the process of applying image recognition technology to the diagnosis of leukemia and malaria in blood smears, the detection and segmentation work are important steps in identifying and analyzing blood cells. The key step is to accurately separate the cells from the background and identify their characteristics [6]. This objective can be attained using segmentation methods like the local adaptive threshold technique. This method adjusts the threshold automatically, enhancing the separation of malaria parasites, thereby improving diagnostic accuracy by addressing the local unevenness of illumination in blood smears [101]. Dahou Yang et al. used the local adaptive threshold method for segmentation, achieving real-time computation speed for segmenting large images, and its robustness ensured accuracy. The segmentation of infected red blood cells (iRBCs) can estimate the number of iRBCs and classify different developmental stages of parasites based on the occupied pixel size [95].

The level set method is a mathematical model that can dynamically evolve contour lines to more accurately capture complex and irregular cell shapes [102]. This method can be used to reasonably segment images based on cell morphological features. Dhanya Bibin et al. used the level set method for segmentation of objects in blood smear images, successfully segmenting peripheral blood smear (PBS) images with complex topological structures [103, 104].

Based on the entropy-based mixture model for fine segmentation using entropy information from images, Krishna Kumar Jha et al. proposed a leukemia automatic detection method called chronological Sine Cosine Algorithm-based actor-critic neural network (Chrono-SCA-ACNN) based on the temporal sine cosine algorithm (SCA). This algorithm utilizes the proposed entropy-based mixture model to segment blood smear images and extract image-level features and statistical features. The model adopts a method that combines fuzzy C-means (FCM) and active contour to segment the fused cytoplasm and nucleus [93].

A rapid segmentation method for white blood cell nuclei based on the differences in RGB color space components contributes to the rapid localization and segmentation of white blood cell nuclei [105]. Experimental results from Wang Yapin demonstrate that the rapid segmentation method based on the differences in RGB color space components performs well in segmenting five types of white blood cells and has a fast segmentation speed. This method is highly suitable for real-time automatic scanning and detection of PBSs [106].On the other hand, the entropy-based mixture model can help determine the optimal segmentation threshold and improve cell recognition and classification [93]. Color space transformations (such as LUV, HSV, HSI) and adaptive thresholding aid in highlighting specific features of cells and simplifying the segmentation task. Das Biplab Kanti et al. successfully segmented blast cells by employing LUV color transformation and adaptive thresholding [100]. For malaria diagnosis, a segmentation method based on color thresholds in the HSV color space can better identify and segment red blood cells with color changes due to malarial parasite infection, thereby improving the early diagnosis accuracy of malaria [99]. Watershed transformation and level set methods are applicable for segmenting cells in contact with each other and cells in complex backgrounds [51, 104]. Additionally, watershed transformation helps address cell adhesion issues, particularly crucial for segmenting overlapping cells and identifying parasite nuclei. Maity Maitreya's experiments indicate that after color normalization and thresholding of digital microscope blood smear images, the watershed algorithm is used to segment red blood cells from the background image. Extracting shape features from the segmented image helps detect shape abnormalities present in microscopic blood smear images, and when this method is followed by feature classification, the classification accuracy and stability are improved [107]. Das Dev Kumar et al. also successfully segmented red blood cells using marker-controlled watershed transformation [94].

Deep learning technologies such as Mask R-CNN, ResNet50, U-Net, and Faster R-CNN can achieve high-precision detection and segmentation by training on large amounts of data to learn complex patterns. This capability is vital for accurately identifying and quantifying leukemia cells or malarial parasites. [108–110].

Furthermore, a combination of various segmentation techniques can be used for image segmentation. Angel Molina employed a method combining threshold segmentation and watershed transformation techniques to segment images, using transfer learning to train the VGG-16 architecture. This model achieved an accuracy of 99.5% in classifying malarial parasites and other red blood cell inclusions, with sensitivity and specificity of 100% and 91.7% respectively, enabling the division of a complete smear into infected or uninfected areas [111]. Additionally, an active contour model combined with a hybrid model of fuzzy C-means algorithm was used to dynamically depict and adjust cell boundaries, accurately capturing cell edges and aiding in distinguishing the irregular shapes of leukemia cells [112].

### Feature extraction and selection

When applying image recognition technology to the diagnosis of leukemia and malaria in blood smears, feature extraction and selection are pivotal steps, with each method serving a distinct role [113].

Color constitutes an essential feature extracted for the diagnosis of leukemia and malaria in blood smears using image recognition technology [113, 114]. Common methods for extracting color features include color histograms and color decomposition [112, 115]. Color histograms are crucial for discerning color variations between normal and abnormal blood cells. Meanwhile, color decomposition methods aid in better identification and analysis of cellular structure and tissue composition by segregating the color components in the blood smear images.

Morphological and textural features of cells can also serve as distinctive features for input into classifiers for classification. Utilizing texture features based on local gradient patterns (LGP) and custom image filtering methods to accentuate specific features, such as edges or specific textures, contributes to the identification of abnormal cell morphology in leukemia. Das Biplab Kanti employed texture features based on LGP and pixel threshold-based methods for blast cell counting [100]. Deep learning and machine learning models hold significant importance in morphological feature extraction. The deep learning model CNN is a potent feature learning tool capable of automatically extracting high-level abstract features from blood smear images, providing a notable advantage in the identification of complex lesions. CNN-based feature extraction can automatically identify and learn morphological and textural features helpful for the diagnosis of leukemia and malaria, facilitating the rapid and automated extraction of required distinctive features [114, 116, 117]. Das Dev Kumar et al. employed machine learning techniques to establish a computer-aided method for the characterization and classification of malaria parasite features in PBS images. They extracted a total of 96 features describing the shape, size, and texture of red blood cells relative to infected and uninfected cells [94].

Furthermore, spectral imaging and image processing techniques can be utilized for feature extraction from blood smears [118]. Raman imaging is an unlabelled spectral imaging technique that can provide information on cellular chemical composition, which is particularly important for detecting abnormal metabolic activity and structural changes in cells. It can offer detailed chemical information at the cellular level, aiding in the differentiation of different cell types and conditions. Additionally, being an unlabelled method, it can avoid staining or processing of samples, thereby reducing potential interference factors. By utilizing this method, faster and feasible cellular feature extraction methods are developed, providing an objective and automated means for disease diagnosis and classification [54].

In addition to the commonly used individual feature extraction methods, different feature extraction methods can also be combined. The entropy-based mixture model combines statistical features and color histogram-based feature extraction methods to extract complexity and irregularity information from images, aiding in the discovery of atypical patterns of leukemia cells [93]. The combination model of degree convolutional autoencoder (DCAE) and CNN can effectively learn deep feature representations from original images, aiding in the detection of subtle pathological features [52]. The joint feature extraction method for color and texture can simultaneously consider the color and texture information of the image, providing a more comprehensive diagnostic basis. Dhanya Bibin et al. completed feature extraction and classification training for deep belief network (DBN) based on connected features of color (histogram-based features and color coherence vector) and texture (Haralick features, local binary pattern features, and gray level co-occurrence matrix features (GLCM)). Finally, DBN was used for the classification of malaria parasites [104]. The hybrid feature engineering method combines the deep learning ability of GoogleNet convolutional neural network with the optimization selection ability of principal component analysis(PCA) and particle swarm optimization(PSO) algorithms, accurately selecting features contributing to the diagnosis of leukemia [119]. The combination of image processing techniques and machine learning algorithms can achieve automated feature extraction and classification, significantly improving diagnostic efficiency and accuracy [120]. In addition to these combined models, there are feature extraction models specifically designed for leukemia, such as the LeuFeatx model. This model, by constructing a fine-tuned feature extraction model compatible with VGG16 specifically for the microscopic images of single leukocytes, is capable of accurately extracting various key features of leukemia cells. Compared to other feature extraction techniques, the LeuFeatx model achieves superior classification performance in several aspects, including macro average precision (MAP), macro average recall (MAR), and macro average F1 (MAF1) [53]. Krishna Kumar Jha et al. also proposed a deep CNN classifier based on Temporal SCA for feature extraction to aid in leukemia diagnosis. This model integrates the segmentation results of the active contour model with the fuzzy C-means algorithm, extracting statistical features and local directional pattern (LDP) features from the segmented images [112].

### Assessment and classification

When applying image recognition technology to the classification of leukemia and malaria in blood smears, a variety of advanced machine learning and deep learning models, or their combinations, can be utilized to enhance accuracy and efficiency. Initially, CNN and their variations, such as Bayesian optimization-based CNN, Chrono-SCA-ACNN, and DCAE-CNN, can be employed to extract and learn image features; these models enhance recognition accuracy through their automated feature learning capabilities. The Bayesian optimization technique employs an informed iterative process to search the hyperparameter space, aiming to find the optimal set of network hyperparameters that minimize the objective error function. Atteia Ghada et al. customized the architecture and hyperparameters of CNN using Bayesian optimization-based methods for input data, demonstrating the superior ALL classification performance of the optimal CNN model based on Bayesian search [121]. Krishna Kumar Jha et al. found that utilizing a deep CNN classifier based on the temporal SCA for classification resulted in higher true positive rate (TPR), true negative rate (TNR), and accuracy compared to other existing techniques, thereby improving the accuracy and reliability of leukemia cell detection and classification [112]. The DCAE-CNN model developed by Tusneem A. Elhassan et al. exhibits high accuracy and sensitivity in detecting various categories of atypical white blood cells, facilitating rapid identification of leukemia cells [52]. In addition to CNN, other deep models such as YOLOv7 and DBNs can also be applied to blood smear recognition. The YOLOv7 model is a real-time object detection system that can perform boundary box prediction and category prediction simultaneously in a single network pass, and can also be adjusted for blood smear image analysis [113]. On the other hand, the DBN, a probabilistic generative model, is composed of stacked layers of restricted Boltzmann machines (RBM) with connections between the layers but no connections between internal units. Dhanya Bibin et al. proposed the use of DBN to identify malarial parasites in human PBS images, marking the first application of this model in malaria detection [104].

Various machine learning algorithms such as support vector machine (SVM) and GFNB can be employed for classification tasks, offering advantages in handling linearly inseparable data and uncertainty [100, 119]. Dahou Yang et al. chose the SVM classifier with a linear kernel to effectively automate the classification of parasitic and non-parasitic images [95]. Das Biplab Kanti utilized the GFNB classifier based on Gini Index, a method for leukemia detection that combines the Gini Index with the Fuzzy Naive Bayes classifier, successfully performing cell counting by employing adaptive threshold segmentation of blast cells. Their approach's reliability was confirmed through experimental analysis using the ALL-IDB1 database [100]. Additionally, ensemble learning models such as random forest (RF), extremely trees classifier (ETC), and extreme gradient boosting (XGB) can be employed to enhance the robustness and generalization capability of the model. These models improve overall performance by combining predictions from multiple weak classifiers [26, 53, 122]

Integration of deep learning and machine learning methods can offer a more comprehensive and powerful solution. For instance, in the integration of deep learning and machine learning methods, the combination of Bayesian learning and SVM is an important strategy. Bayesian learning can model uncertainty, while SVM excels in handling linearly inseparable data and classification. By combining these two methods, the recognition of blood smear images can be more comprehensive, and diagnostic accuracy can be improved. Das Dev Kumar et al. established a computer-aided method for malaria parasite feature extraction and classification using machine learning methods based on PBS images. They designed a feature selection and classification scheme by combining F-statistic, statistical learning techniques, i.e., Bayesian learning and support vector machine, to utilize the optimal discriminative feature set for higher classification accuracy [94]. Angel Molina et al. developed a machine learning method that can not only distinguish parasitized red blood cells from normal red blood cells but also differentiate other red blood cell inclusions, such as Howell-Jolly and Pappenheimer bodies, basophilic stippling, and platelets on red blood cells [122]. They further developed the first deep learning model combined with a convolutional neural network, which can distinguish iRBCs from normal red blood cells as well as differentiate red blood cells from other types of inclusions [111]. WT-DFN, as a weakly supervised data augmentation classification network based on ternary flow, integrates data augmentation deep learning techniques and weakly supervised learning machine learning strategies to enhance the model's performance in situations with limited annotated data. Through this hybrid approach, we can leverage the advantages of deep learning in feature extraction while combining the strong capabilities of machine learning algorithms in classification and generalization to further improve the diagnostic accuracy of blood smear images [26].

Overall, the combined use of these models can provide a comprehensive framework for automatically extracting features, classifying, and ultimately diagnosing leukemia and malaria from blood smear images. By integrating the strengths of these models, we can design a powerful diagnostic system that offers high-precision, efficient medical diagnostic support.

With the advancement of smartphone technology, numerous scholars have embarked on research integrating these portable devices with blood diagnostics. Rosado et al. proposed an image processing and analysis methodology employing supervised classification to assess the presence of malignant Plasmodium falciparum trophozoites and leukocytes in Giemsa-stained thick blood smears. Utilizing support vector machines along with a blend of geometric, color, and texture features, they achieved an automatic detection sensitivity of 80.5% and specificity of 93.8% for trophozoites, while for leukocytes, the sensitivity was 98.2% with a specificity of 72.1% [123].

**Fig. 1:**
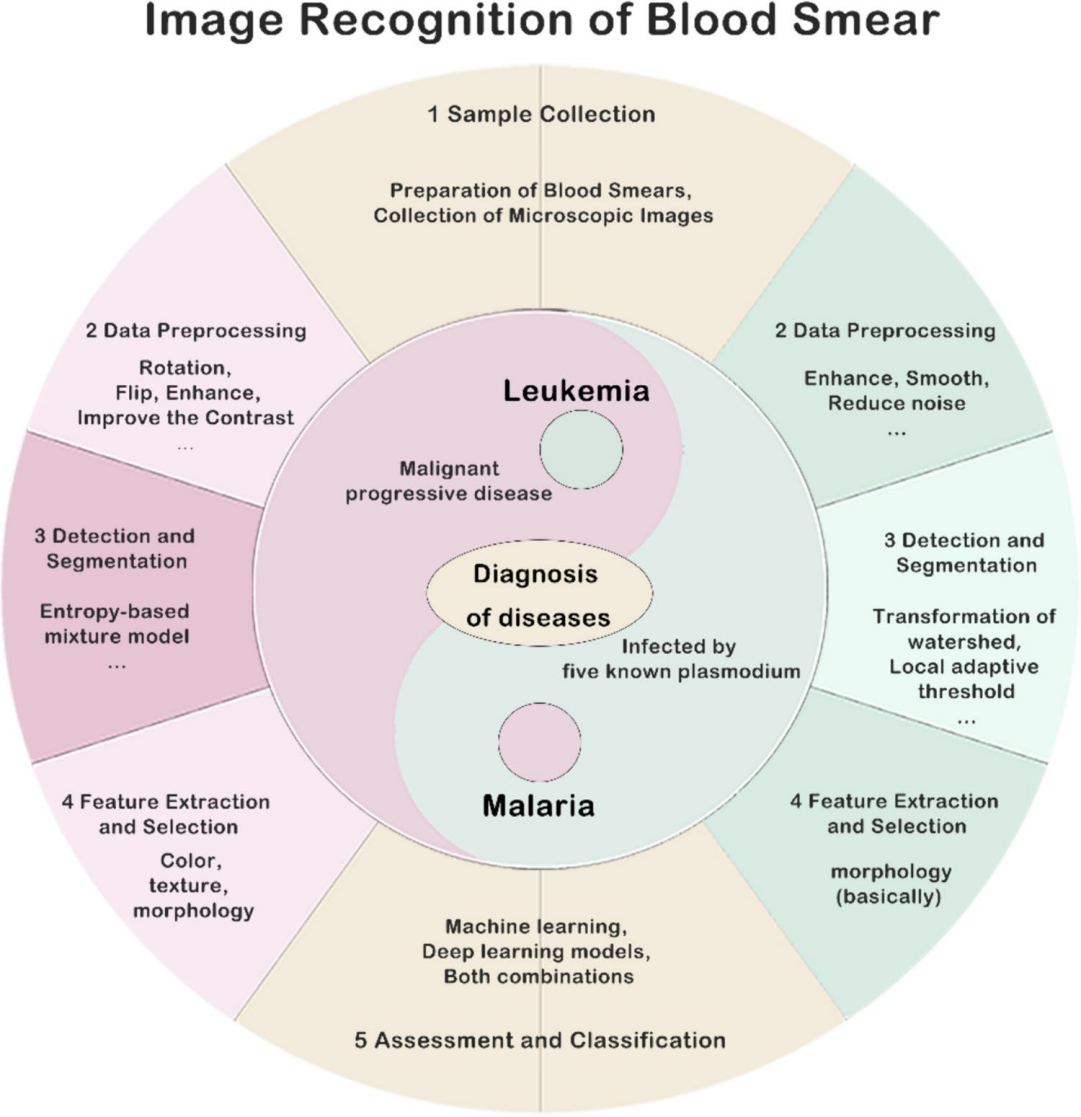
Summary of the application steps of image recognition technology in blood smear analysis (focusing on leukemia and malaria). The figure delineates the sequential steps and both the similarities and differences in the application of image recognition technology for the detection of leukemia and malaria in blood smears

**Fig. 2:**
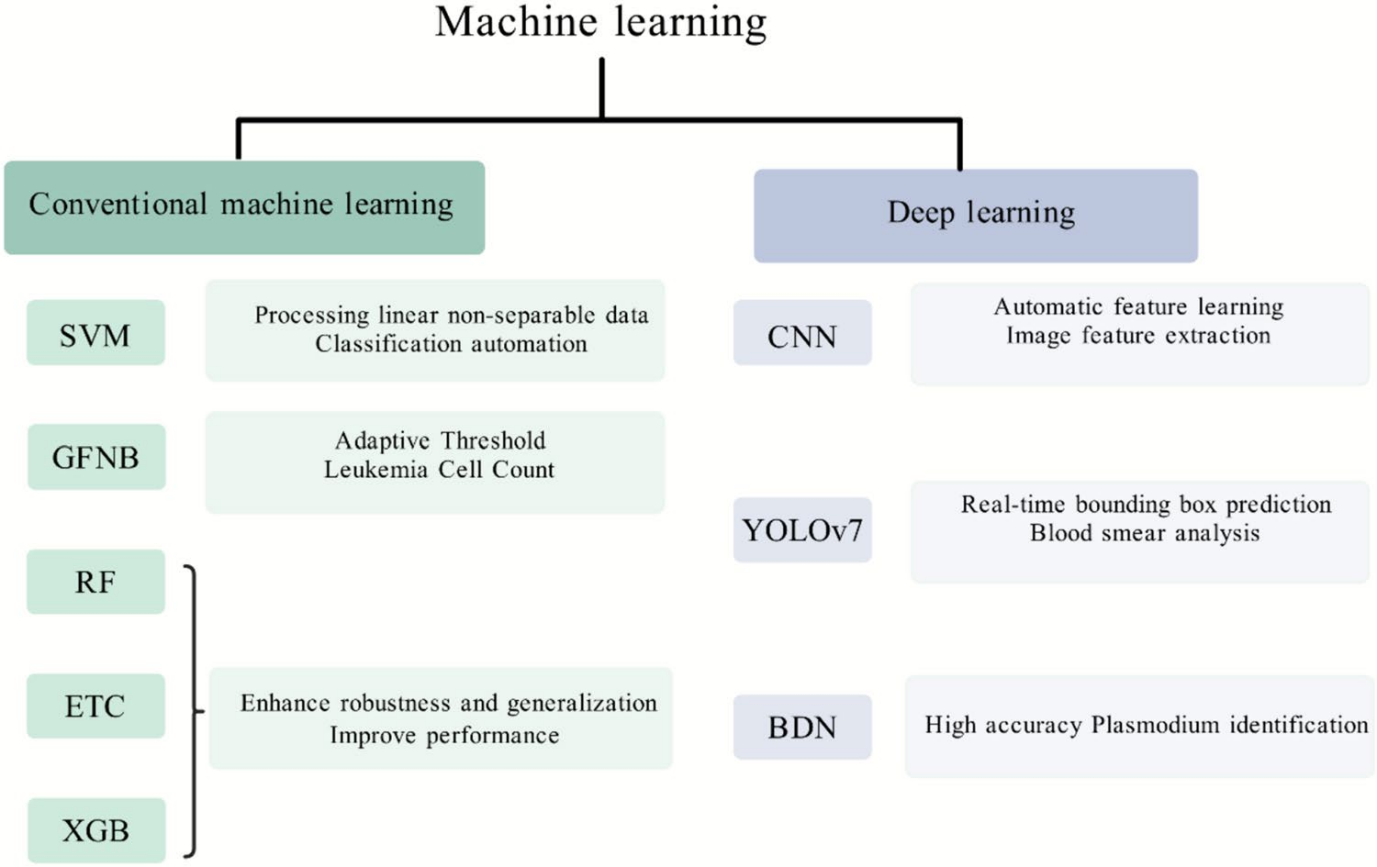
Summary of major machine learning algorithms. This image provides an overview of the various machine learning techniques discussed in the article, offering a categorical summary. It outlines their applications and benefits specifically in the context of blood smear image recognition. SVM: support vector machine, GFNB: Gini index-based Fuzzy Naive Bayes classifier, RF: random forest, ETC: extremely trees classifier, XGB: extreme gradient boosting, CNN: convolutional neural networks, BDN: deep belief network

**Fig. 3:**
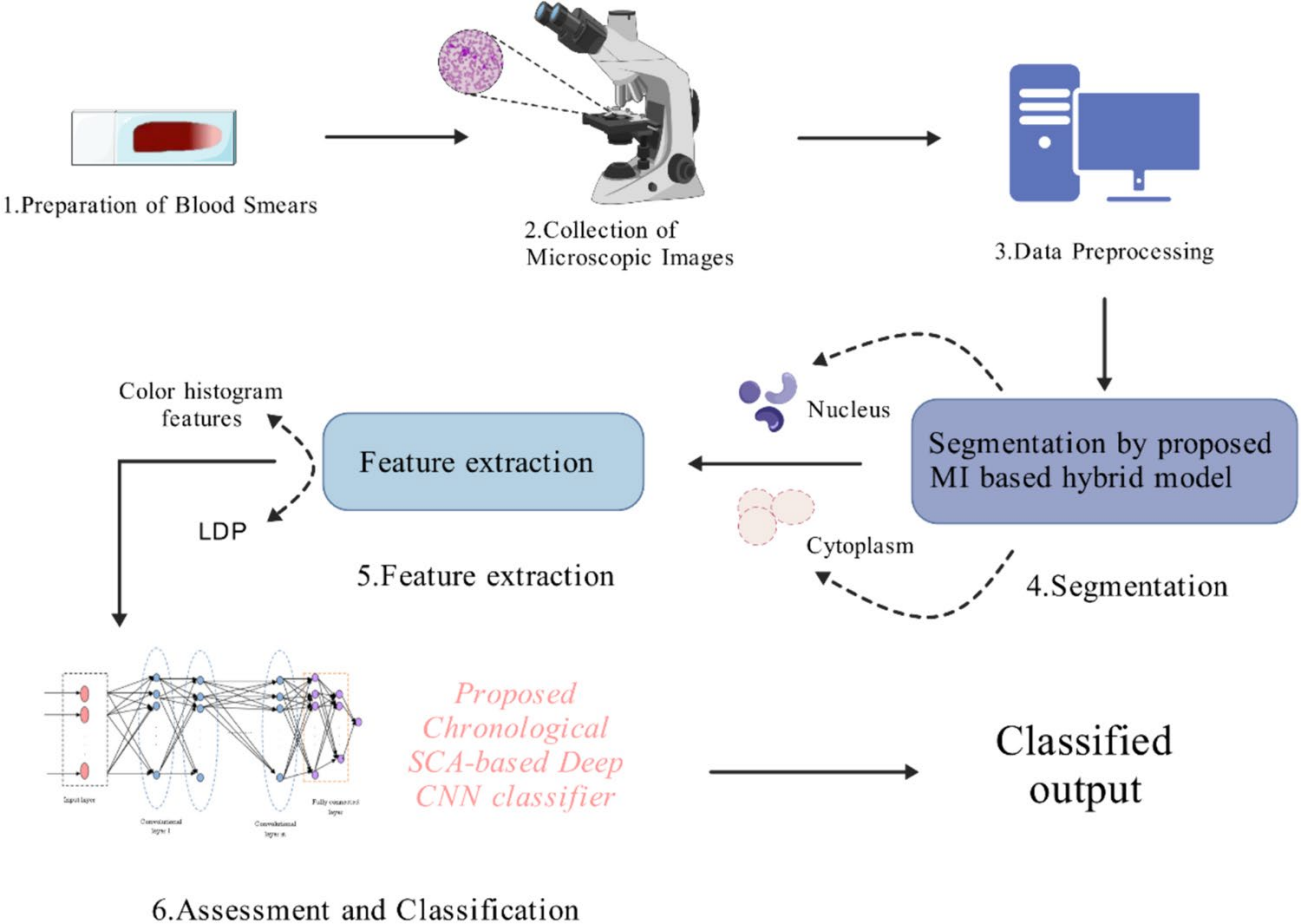
An example of a workflow applying image recognition technology. This image summarizes an important workflow by K.K. Jha and H.S. Dutta for the diagnosis of acute lymphoblastic leukemia using image recognition technology

Dallet et al. described a mobile application platform for Android smartphones capable of diagnosing malaria from Giemsa-stained thin blood film images. The platform's primary imaging components include sophisticated morphological operations that can detect erythrocytes and leukocytes and identify parasites within infected cells. The application also can distinguish different life stages of the parasite and calculate the level of parasitemia [124] (Figs. 1, 2, 3).

## Practical applications

### Intelligent diagnosis of leukemia

The quantity of platelets (PLT) is an important indicator for diagnosing leukemia. Currently, each PLT counting technique has its specific inaccuracies and lacks a unified standard [125], which could lead to significant technical errors in the diagnosis of leukemia. Presently, the four main methods for platelet counting include manual phase-contrast microscopy, impedance, optical light scattering/fluorescence, and flow cytometry [126].

Sysmex, as a well-established brand of clinical hematology analyzers, has developed multiple series tailored to different users and purposes. Among them, the Sysmex XN-9000, the flagship model in the XN series, includes analysis modules, slide makers/stainers, and digital imaging modules, and it can perform PLT testing using three methods: impedance channel (PLT-I), light channel (PLT-O), and fluorescence channel (PLT-F). Testing has shown excellent consistency in samples with reduced platelets, with better average differences, correlations, and consistency demonstrated in PLT-F [127]. Additionally, the PLT-F of Sysmex XN-2000 has also been proven to be sufficiently accurate [125]. Both of these are relatively advantages for the diagnosis of leukemia.

Mindray Medical's BC-6800, utilizing unique three-dimensional cell analysis technology, automatically increases the analysis volume for low-value samples, thus enhancing the accuracy of blood cell classification through the analysis of cell particle forward light scatter signals, side scatter light signals, and fluorescence signals. Its subsidiary BC-6000 Plus, based on the principle of optical counting, was found in a comparative experiment to have better accuracy and repeatability in platelet counting in samples with reduced platelets compared to Sysmex XN-9000's PLT-F, making it a reliable choice for PLT counting [127]. According to findings from another comparative experiment, the BC-6800 Plus shows very small average differences from the International Society for Laboratory Hematology (ISLH) reference method (IRM), especially in the counting of normal platelet samples, demonstrating remarkable correlations and consistency [128], and it can also serve as a reference for the diagnosis of leukemia.

The accuracy of CellaVision's DM9600 system in identifying and classifying white blood cells and red blood cells has been validated in multiple experiments [129–131]. There are even teams that have used this system to collect and create the first publicly available large collection of eight classes of normal peripheral blood cells, making it a potential standard dataset for benchmark testing models [132], providing a reference standard for the automated diagnosis of leukemia using machine learning.

### Intelligent diagnosis of malaria

Microscopic examination remains the gold standard for the laboratory diagnosis of malaria. Blood samples collected from patients are prepared as thick or thin blood smears and stained using Romanovsky staining agents. However, routine performance of this test by laboratory personnel is not guaranteed, hence proficiency in the technique may not be ensured [133].

The team led by Stefan Jaeger developed an Android smartphone application for malaria screening, known as Malaria Screener. Its source code is hosted on GitHub as an open-source project (https://github.com/nlm-malaria/MalariaScreener), allowing for further modification and development. The software comprises three primary functional modules: the slide screening module (image acquisition, parasite detection, and result visualization), data management module, and data upload module [134]. Through deep learning with manually annotated training images, the software is capable of autonomously locating and enumerating parasites in both thin and thick smears. For thin smears, a dual deep learning architecture for cell clustering, termed RBCNet, is employed to detect and count parasites within red blood cells. This includes a U-Net for cell cluster or superpixel segmentation and a Faster R-CNN to refine the detection of small cell objects within connected component clusters [110]. For thick smears, the current approach uses the Iterative Global Minimum Screening (IGMS) method for rapid preliminary selection of parasites, followed by parasite classification using a newly developed CNN model (comprising seven convolutional layers, three max-pooling layers, three fully connected layers, and a softmax layer), all implemented using the OpenCV4Android SDK library [135]. However, its use is limited to the detection of Plasmodium falciparum. Consequently, developers are researching a new framework capable of detecting and differentiating between Plasmodium falciparum and Plasmodium vivax, tentatively not yet updated to Malaria Screener: PlasmodiumVF-Net. This employs a Mask R-CNN trained on three classes to detect malaria parasites, coupled with a ResNet50 classifier to filter out false positives, followed by classification based on the quantity and aggregated probability of patches detected across all patient images [108]. The software has demonstrated a level of advancement and superiority through evaluations [108, 136]

The Sysmex XN-1000, as an adjunct to PBS examination or manual platelet count difference analysis (MPDA), is employed for the analysis of hematological parameter data, leukocyte cell population data (LCPD), associated scattergrams, and forward scatter (FCS) data. It has demonstrated notable efficacy in the detection of malaria. The results indicate that the AUC for Δwhite blood cell(WBC) [(WBC-D) – (WBC-N)] in the malaria-positive group is 0.9364, with a sensitivity of 85.42% (95% CI 72.24–93.93%) and a specificity of 88.16% (95% CI 86.22–89.91%), evidencing its excellent accuracy as a supportive basis for malaria diagnosis [137]. Similarly, the XN-30 analyzer precisely identifies and quantifies the total developmental stages of cultured Plasmodium falciparum and each stage automatically, based on DNA content and cell size, with results exhibiting high accuracy and repeatability [138].

The EasyScan GO, co-developed by Motic and Global Health Labs, is a microscope device that utilizes machine learning-based image analysis [139]. In its most recent evaluation, the EasyScan GO demonstrated a diagnostic sensitivity of 91.1% (95% CI 88.9–92.7) and a specificity of 75.6% (95% CI 73.1–78.0) for Giemsa-stained blood smears, with both sensitivity and specificity improving as the quality of the slide preparation increased. Its performance in parasite detection and species identification accuracy meets the World Health Organization’s Special Programme for Research and Training in Tropical Diseases (WHO-TDR) level 2 standard for malaria microscopy capability. However, the performance parameters were greatly influenced by the quality of the slide preparation [140].

As the first artificial intelligence-based high-resolution full-field morphology analyzer for peripheral blood specimens, Scopio Labs’ X100 boasts a physics-based reconstruction model that calculates clear full-field PBS images with resolution capabilities up to 100X. It also features an AI-driven decision support system. The system not only automates the detection of white blood cells to analyze and pre-classify 200 white blood cells into 16 categories but also leverages full-field view technology for PBS, which is particularly conducive to assessing the extent of red blood cell anomalies, such as the presence of malaria trophozoites [141].

A preliminary experimental evaluation showed that the advanced red blood cell(RBC) application within the CellaVision DM9600 system, although demonstrating low sensitivity (23.5%) for the detection of red blood cell inclusions, rendering it unsuitable for malaria screening, may still be beneficial for the follow-up of malaria due to its strong correlation with microscopic examination and reduced turnaround time [13, 142]

The automatic whole slide scanner AI100 with the Shonit™ system is capable of scanning slides, automatically quantifying the morphology of WBCs, RBCs, and platelets and their subtypes based on deep learning, and performing classification. It has been tested to have a sensitivity and specificity of up to 91% and over 98% [143]. Additionally, it is compatible with all Romanowsky stains [144]. The classification and counting of various subtypes of white blood cells by the AI100 instrument can essentially replace the qualitative analysis of PBS smears based on manual microscopy. in future, the morphological information of white blood cells based on PBS automatic analysis is expected to be used for the diagnosis of leukemia. Currently, the AI100 has also been widely used in various fields: to identify COVID-19 positive or negative status [145], to assist in the weakly supervised detection of diabetic retinopathy abnormalities [146], and to complete imaging-based Sodium Lauryl Sulfate (SLS) measurements on microfluidic chips to quantify SLS-hemoglobin complexes' Soret peak, achieving the diagnosis of anemia [147].

In addition to the above, there are also artificial intelligence devices (or software) for auxiliary diagnosis, such as the AI-based microscope mobile application Celly for iPhone [148] and the machine learning-based application design provided by MicroscopeIT for image segmentation, object detection, data processing, and management [149].

## Discussion

Numerous scholars are currently exploring various machine learning image recognition algorithms for pathological detection of diseases such as leukemia and malaria, as previously summarized. The quintessential clinical hematology tests primarily comprise complete blood count (CBC) and PBS. To establish an integrated hematological diagnostic system, a comprehensive blood panel, including RBC, hemoglobin (Hb), WBC, differential leukocyte count, and platelets, is crucial [150]. Also known as a full blood count, this test measures the number and characteristics of blood cells, including red blood cells, white blood cells, platelets, hemoglobin concentration, hematocrit, and mean corpuscular volume (MCV), aiding in the preliminary detection of various conditions such as infections, anemia, immune system disorders, and blood cancers [151, 152]. Compared to the full blood count, the peripheral blood smear offers additional measurements and morphological classifications of white blood cells under a microscope, as well as assessment of red blood cell color, size, shape, and estimation of platelet count [153]. As a follow-up to abnormal full blood count results, it facilitates further investigation into hematological diseases such as leukemia and anemia, bone marrow disorders, and infections/inflammatory conditions [72, 154, 155]. The complete blood count with differential specifically quantifies and categorizes white blood cells on top of the basic full blood count, enabling precise evaluation of risks associated with blood diseases like leukemia, autoimmune disorders, and infections [156].

Despite the significant advantages offered by image recognition technology, it is imperative to acknowledge its potential limitations and challenges. For instance, The precise segmentation of malaria images has been a significant challenge in image recognition technology due to the difficulty in locating the malaria parasites within the cells (malaria parasites belong to the group of intracellular parasites) and the challenge of distinguishing them from other cells (such as normal red blood cells, red blood cells infected by parasites, white blood cells, and platelet clots, which may appear very similar morphologically) [157]. The inconsistency in image quality and the diversity of different microscopic equipment can also impact the performance of algorithms. Furthermore, for complex disease patterns, image recognition algorithms may require a larger sample size for training to ensure high accuracy and reliability [158]. The privacy and security of patient data is another critical aspect that must be taken into consideration [159]. Given that these systems typically require extensive datasets for training, it is essential to maintain patient confidentiality and ensure that the use of data adheres to ethical standards and complies with relevant regulations.

in future, the direction of research should focus on the development of a comprehensive system for the detection of haematopathology. This system will integrate multiple image recognition algorithms to accommodate different types of blood smears and diseases. At the same time, to address the issue of class similarity in traditional image recognition tasks, exploring multi-class classifiers can be a novel direction. For instance, Saxena introduced an innovative ternary classifier approach that overcomes the limitations of previous studies by creating a comprehensive dataset encompassing normal red blood cells, infected red blood cells, white blood cells, and platelets. This advancement has enhanced the accuracy of malaria blood smear identification [157]. Furthermore, research should focus on improving the generalization capabilities of the algorithms so that they can operate effectively across different laboratories and devices. In addition, with the growing concern about the ethical aspects of AI, ensuring that the application of these techniques does not violate patient privacy and safety is also an important aspect of future research.

In conclusion, the application of image recognition technology in blood smear diagnosis is promising, and its ability to improve diagnostic accuracy and efficiency can have a significant impact on improving the diagnostic and therapeutic process for patients. Through continued research and development, we can expect that this field will continue to make progress in future and bring about positive changes in global health.

## Conclusion

In conclusion, the application of image recognition technology driven by neural networks and deep learning has greatly transformed the process of blood smear analysis, providing a more efficient, accurate, and subjective alternative to traditional manual diagnostic methods. By summarizing the application methods and steps of these algorithms, particularly in the diagnosis of malaria and leukemia through blood smear analysis, it has laid the foundation for the further development of rapid diagnosis of these diseases and provided direction for the future diagnosis of other diseases through blood smear analysis. The advancement of computer technology has not only simplified the process of identifying blood cells but has also paved the way for a comprehensive blood cell pathological detection system. This system has the potential to completely change diagnostic methods, alleviate the burden on clinical laboratory analysts, and ultimately improve patient treatment outcomes.
